# The Myocardial Unfolded Protein Response during Ischemic Cardiovascular Disease

**DOI:** 10.1155/2012/583170

**Published:** 2012-03-29

**Authors:** Edward B. Thorp

**Affiliations:** Department of Pathology and Feinberg Cardiovascular Research Institute, Feinberg School of Medicine, Northwestern University, 300 East Superior Street, Tarry Building 3-705, Chicago, IL 60611, USA

## Abstract

Heart failure is a progressive and disabling disease. The incidence of heart failure is also on the rise, particularly in the elderly of industrialized societies. This is in part due to an increased ageing population, whom initially benefits from improved, and life-extending cardiovascular therapy, yet ultimately succumb to myocardial failure. A major cause of heart failure is ischemia secondary to the sequence of events that is dyslipidemia, atherosclerosis, and myocardial infarction. In the case of heart failure postmyocardial infarction, ischemia can lead to myocardial cell death by both necrosis and apoptosis. The extent of myocyte death postinfarction is associated with adverse cardiac remodeling that can contribute to progressive heart chamber dilation, ventricular wall thinning, and the onset of loss of cardiac function. In cardiomyocytes, recent studies indicate that myocardial ischemic injury activates the unfolded protein stress response (UPR) and this is associated with increased apoptosis. This paper focuses on the intersection of ischemia, the UPR, and cell death in cardiomyocytes. Targeting of the myocardial UPR may prove to be a viable target for the prevention of myocyte cell loss and the progression of heart failure due to ischemic injury.

## 1. Introduction

Heart failure (HF) is a common condition and leading cause of hospitalization in the United States and developed countries. HF can be debilitating and lead to reduced cardiac output, physical disability, and mortality. The numbers of HF cases in the USA are increasing, in line with a rise in the elderly population who are at increased risk [1, 2]. Common causes of HF include ischemic heart disease (including myocardial infarction), hypertension, cardiomyopathy, and valvular heart disease. In the case of ischemic heart disease and myocardial infarction (MI), advances in patient care have reduced the risk of susceptibility to MI and of immediate death. Thus, while there has been an increase in the numbers who initially survive an acute MI, this improvement has been offset by more survivors progressing to HF [3]. This deterioration often leads to left ventricular systolic dysfunction and can be linked to the initial cardiac damage and remodeling early after myocardial ischemia. Thus, new therapeutic targets and treatments are needed to combat the morbidity and mortality caused after MI-induced HF.

### 1.1. Cardiomyocyte Death in HF

 To date, the failure of a heart to deliver blood that is sufficient for the metabolic needs of the body is largely irreversible. Loss of cardiomyocytes by cell death contributes to reduced cardiac output. In the case of myocardial infarction, acute ischemia can lead to significant levels of cardiomyocyte death. Myocardial ischemia after MI is a significant cellular stress that promotes cardiomyocyte death by either necrosis or apoptosis [4]. In patients, increased myocardial apoptosis has been associated with unfavorable ventricular remodeling and early symptoms of post MI heart failure [5]. Adverse cardiac remodeling involves scar and fibrous tissue formation, whereby the chambers of the heart enlarge and contractility become less efficient [6]. At the cellular level, death of the cardiomyocyte depends on the duration of ischemia and also on the capacity of the myocyte to respond to the ischemic stress. Numerous cellular responses have been identified in cardiomyocytes under ischemic stress and HF. For example, autophagy is activated in HF and may suppress hypertrophy through increased protein degradation [7]. Accumulating evidence indicates that another significant stress response in cardiomyocytes can affect cell survival. During ischemia, the unfolded protein response (UPR) or integrated stress response is activated in myocytes, as described below.

### 1.2. The UPR

In noncardiac cells, the UPR signals from the endoplasmic reticulum [8], which is responsible for the synthesis and folding of proteins, as well as calcium storage and other signaling pathways. Under conditions that perturb endoplasmic reticulum homeostasis, the ER has the capacity to adapt and activate the UPR to compensate and attempt to restore organelle equilibrium [8]. The function of the UPR is to protect the ER from normal and pathophysiological perturbations in development and disease that include elevated protein synthesis, disruption of ER calcium homeostasis, changes in redox potential, and disturbances in the physical properties of the ER membrane bilayer [9, 10]. The UPR is composed of three main signaling branches. These include inositol-requiring enzyme-1 (IRE-1) [11] activating transcription factor-6 (ATF6) [12] and PKR-like eukaryotic initiation factor 2 kinase (PERK) [13]. Activation of the UPR regulates multiple compensatory gene expression pathways, including induction of protein-folding chaperones, phospholipid biosynthesis, oxidoreductases, and the promotion of terminally misfolded protein degradation, through the ER-associated degradation pathway (ERAD) [8, 14, 15]. The UPR also exerts translational control by phosphorylating the eukaryotic initiating factor eIF2*α* and selectively reduces protein translation to lessen the load on the ER [16]. These compensatory pathways act first in an attempt to reconstitute cell and ER homeostasis. If homeostasis is restored, this induces a negative feedback of the UPR [17]. If disequilibrium persists, proapoptotic pathways can be induced [18], as discussed below.

### 1.3. SR/ER and the UPR

 Within the cardiomyocyte, the sarcoplasmic reticulum (SR) is a specialized endoplasmic reticulum and extensive network within the cell that regulates calcium (Ca^2+^) flux and excitation contraction coupling. Under conditions of heart disease, the SR is expanded, consistent with a compensatory response to stress [19]. Through the years, the terms sarcoplasmic reticulum and endoplasmic reticulum have been used interchangeably. Indeed, numerous canonical ER proteins, including protein chaperones, can be found in myocytes after relatively crude biochemical fractionations of the SR. Such ER proteins that have been identified in cardiac tissue include Bip, Grp94, calnexin, PDI, and others [20–22]. Cardiomyocytes, like other cells, require these proteins and chaperones to promote protein folding and other housekeeping functions synonymous with the ER. In addition to encoding canonical ER-resident proteins, cardiomyocytes can also activate the UPR in response to characteristic UPR inducers, such as protein-folding disequilibrium. For example, the Lys-Asp-Glu-Leu (KDEL) receptor, an ER retrieval receptor for protein chaperones, promotes chaperone accumulation in the ER/early secretory pathway. In an experimental model of forced gene activation, transgenic expression of a dysfunctional KDEL receptor induced UPR markers in myocardial tissue [23]. Such protein-folding disorders in the heart have also been linked to cardiomyocyte death, as transgenic overexpression of preamyloid oligomers induces apoptosis in cardiomyocytes [24]. In another example of myocardial protein dysregulation, a R120G mutation in CryAB (crystallin, alpha B), a small heat shock protein, is linked to familial cardiomyopathy. This mutation induces CryAB protein aggregation and in mice, overexpression of R120G mutant CryAB induces cardiomyopathy, whereas overexpression of its wild-type counterpart does not [25]. Furthermore, conditions of increased protein synthesis, such as during hypertrophy, appear to activate the UPR [26]. Some have interestingly suggested that the SR and ER are spatially and functionally distinct [27, 28]. Regardless of this distinction, cardiomyocyte stress induces the UPR, and conditions that can adversely affect protein folding, similar to in noncardiomyocytes, are toxic in the myocardium and linked to activation of UPR pathways.

### 1.4. Ischemic Stress

 In experimental models of myocardial ischemia, activation of UPR chaperones has been shown to occur during development of ischemic heart disease [29]. Ischemia is a major contributor to heart failure, and the reduction in supply of oxygen to the heart is a significant stress on myocardial tissue. Even prior to myocardial infarction, expanding atherosclerotic plaque in coronary arteries reduces blood flow and oxygen in downstream coronary tissue. Loss of perfusion leads to a drop in oxygen and a transition to glycolytic energy production. Ischemic myocardium is characterized by reduced oxidative phosphorylation and increased anaerobic metabolism [30]. Reliance on glycolysis and accumulation of inorganic phosphate also lead to cellular acidification through increases in lactic acid production [31]. These factors in combination can significantly compromise cellular energy production by reducing generation of adenosine triphosphate (ATP). Ischemia also contributes to mitochondria dysfunction. In heart cells, mitochondria swell and release cytochrome C, contributing to contractile dysfunction [32]. When prolonged, ischemia will promote caspase-mediated apoptosis in cardiomyocytes. *In vitro*, ischemia can be simulated through deprivation of serum, glucose, and oxygen (SGO). Ischemia, in other tissues, has been shown to lead to the impairment of protein folding in the ER, leading to activation of the UPR. Hypoxia alone leads to dysfunctional disulfide bond generation by oxygen-dependent protein disulfide isomerase and this in turn leads to protein misfolding and activation of the UPR [33]. Reoxygenation effects on the UPR in cardiomyocytes also are a significant factor [34]. Below, we highlight how ischemia can lead to modulation of the UPR in the heart. Ischemia has been linked to the activation of all three arms of the UPR as described next.

## 2. The Cardiomyocyte UPR

### 2.1. The Cardiomyocyte IRE-1*α* Pathway and Ischemia

**Figure 1:**
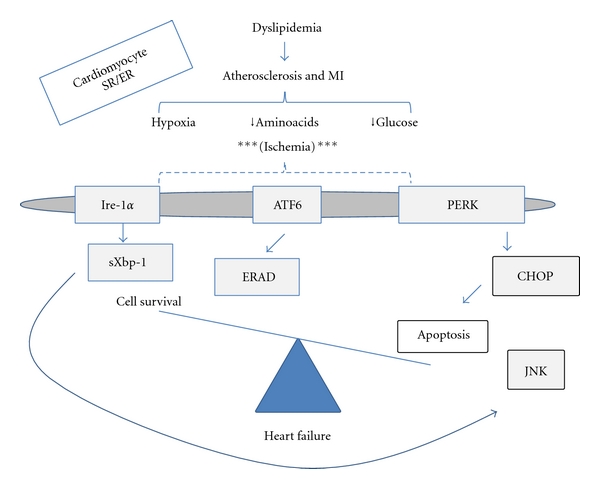
Working model of the cardiomyocyte unfolded protein response during ischemia. Activators of the UPR in cardiomyocytes at the sarcoplasmic reticulum (SR)/endoplasmic reticulu (ER) include ischemia during cardiovascular disease. Proximal effectors of the UPR include IRE-1*α*, ATF6, and PERK. IRE-1*α* induces splicing of *Xbp-1* mRNA and can promote prosurvival or proapoptotic pathways. ATF6 proteolysis leads to transcription of ER stress responsive genes and has been implicated in cardiomyocyte cell survival. Initial effects of PERK include translational arrest that reduces the load on the SR/ER folding machinery. Downstream and distal effector responses of PERK include CHOP, which promotes cardiomyocyte apoptosis and may contribute to heart failure.

The ER transmembrane protein IRE-1*α* is homodimerized during ER stress to induce autophosphorylation. Homodimerization is induced by sequestration of GRP78/Bip through an accumulation of misfolded protein in the ER [35]. ER stress also activates an IRE-1*α* endoribonuclease activity that splices X-box-binding protein-1 (*Xbp-1*) mRNA (Figure 1). Spliced *Xbp-1* (*sXbp-1*) encodes a basic leucine-zipper and active form of XBP-1, which induces ER stress response genes [36, 37] that escape PERK-mediated translational arrest (discussed below). The requirement for the UPR in the heart and the IRE-1*α* pathway begins during embryonic development. GRP78 (guanine-nucleotide-releasing protein 78), an IRE-1*α* and ATF6 downstream target, is upregulated in the embryonic mouse myocardium. In addition, *Xbp-1* is required for heart formation as *Xbp-1* deficient mice die in utero. *Xbp-1* knockout mice death occurs in association with significant cardiomyocyte death [38]. Consistent with a prosurvival role for XBP-1, inhibition of IRE-1*α* reduces chemokine-induced autophagic cell death in H9c2 cardiomyocytes [39]. In experimental models of ischemia (in hearts post MI), the IRE-1*α* downstream target GRP78 is upregulated in myocardial tissue proximal to the infarct [40]. *Ex vivo* (in a Langendorff heart perfusion system), GRP78, and *sXbp-1* are induced during simulated ischemia and reperfusion [41]. *In vitro*, primary neonatal rat cardiomyocytes exposed to serum, glucose, and oxygen deprivation (SGO) can induce spliced *Xbp-1* mRNA, and this occurs within hours [42]. As evidence for a causal role of XBP-1 during ischemia, adenoviral dominant negative XBP-1 expression resulted in increased hypoxia-reoxygenation-induced apoptosis. The IRE-1*α* pathway has also been implicated in proapoptotic pathways as well. For example, in noncardiomyocytes, IRE-1*α* can interact with the adaptor protein TNF receptor-associated factor (TRAF2). IRE-1*α* and TRAF2 subsequently act on ASK1 (mitogen-activated protein kinase kinase kinase), which phosphorylates proapoptotic JNK [43]. Less is known regarding how such a proapoptotic IRE-1*α* pathway may function in cardiomyocytes. In addition, calcium dysregulation is an important component of ischemic heart failure and upregulation of sarco/endoplasmic reticulum calcium-ATPase isoform 3f (SERCA3f) is associated with heart failure [44]. Experimental overexpression of SERCA3f has been shown to induce *Xbp-1* splicing. Also, cardiomyocyte-specific disruption of the calcium regulator *Serca2* induces the UPR and promotes apoptosis [44]. Overexpression of the downstream target of XBP-1, GRP94 reduced H9c2 cardiomyocyte necrosis induced by both calcium overload and ischemia [45]. Thus, although the aforementioned examples indicate a significant role for the IRE-1*α* pathway in cardiomyocyte survival and during calcium regulation, much remains to be understood, including how the prosurvival roles of IRE-1*α* signaling may differentially act during development versus after ischemic injury.

### 2.2. Activating Transcription Factor 6 (ATF6) in the Heart

On activation of the UPR, ATF6 travels to the Golgi, where its cleavage leads to the translocation of its cytosolic fragment to the nucleus and binding to ER stress response elements (ERSEs). Cleaved ATF6 then promotes transcription of ER-targeted genes, such as the ER chaperone, GRP78. In mice after MI, inhibition of ATF6 activation with 4-(2-aminoethyl) benzenesulfonyl fluoride, an inhibitor of ATF6, impaired cardiac function and increased mortality. In contrast, cardiac function after MI was improved in mice expressing a constitutively active mutant of *Atf6*, compared with wild-type littermates [46] and consistent with a protective role. In primary murine cardiac myocytes exposed to oxygen and nutrient deprivation, membrane-associated ATF6 was reduced with a concomitant increase in nuclear ATF6 [47]. This ischemia-induced event was accompanied by ATF6 binding to the ERSE of GRP78, transcriptional upregulation of GRP78 and was reversible by simulated reperfusion *in vitro*. More importantly, a dominant-negative form of ATF6 prevented inducement of Grp78 and promoted cardiomyocyte cell death, indicating a prosurvival role for ATF6. ATF6 has also been shown to induce ER-associated degradation (ERAD). ERAD has been shown to alleviate ER stress by degrading misfolded protein in the ER [15]. Interestingly, Belmont et al. discovered that Derlin-3, a component of ERAD, is induced by ATF6 in the mouse heart [48]. Furthermore, overexpression of Derlin-3 protected cardiomyocytes *in vitro* from simulated ischemia-induced apoptosis. In another article by Belmont, transcriptional profiling indentified *modulatory calcineurin interacting protein-1* (*MCIP1*), also known as *regulator of calcineurin 1* (*RCAN1*), as a novel ATF6-inducible gene. They found that ATF6 was able to induce RCAN1 in cultured cardiac myocytes and that adenoviral overexpression of activated ATF6 further induced RCAN1 and modulated cell growth [49]. Thus, ATF6 is induced under ischemic conditions and can play a role to help protect cardiomyocyte survival. Interestingly, an ATF6 isoform and other ATF6-related proteins may play a role in regulating the UPR, however, their full roles in cardiomyocytes remain undetermined and should be subject of future investigation [50].

### 2.3. Cardiomyocyte PERK (dsRNA-Activated Protein Kinase-Like Endoplasmic Reticulum Kinase)

Though the IRE-1*α* and ATF6 branches have for the most part been associated with prosurvival roles in cardiomyocytes, prolonged activation of the PERK/ATF4/CHOP pathway is principally implicated in cardiomyocyte cell death. Downstream of PERK, phosphorylation of eIF2*α* can be detected as early as one hour after ischemia *in vitro* in cardiomyocytes [42]. Eukaryotic translation initiation factor 2*α* (eIF2*α*) phosphorylation leads to a transient downregulation of the majority of protein synthesis through inhibition of cap-dependent protein translation. Only transcripts encoded by ER stress response genes are induced, reducing the demands on the ER. This may have implications in prevention of cardiac hypertrophy and is part of the initial compensatory pathway of the PERK branch towards promoting survival. Under prolonged ER stress, C/EBP homologous protein (CHOP) is induced. Myocardial tissue from patients with heart failure exhibits increased *Chop* mRNA. Okada et al. reported that prolonged ER stress occurs in hypertrophic and failing hearts after aortic constriction [51]. Also, *Chop* deficiency reduces cardiac apoptosis in a pressure overload model of heart disease [52]. CHOP has also been implicated in dilated cardiomyopathy [23]. *In vitro*, in heart cells, prolonged ER stress induced by ischemia promoted the activation of CHOP [42], processing of procaspase-12 and induction of apoptosis. Consistent with activation by ischemia, *Chop* transcription is also regulated by amino acid starvation. For example, an upstream cis amino acid response element in *Chop* has been found to bind activating transcription factor 2 (ATF-2) and expression of ATF-2 is required for the transcriptional activation of *Chop* by leucine starvation *in vitro* [53]. In support of this pathway being activated during ischemia, ATF-2 is stabilized by hypoxia [54]. More recently, prostatic androgen repressed message-1 or PARM was identified to be predominantly expressed in cardiomyocytes and a negative regulator of CHOP-mediated apoptosis [55]. Finally, *Chop* deficiency has been shown to reduce myocardial reperfusion injury in a mouse model of MI [56]. Future studies *in vivo* are warranted to separate the effects of CHOP after ischemia as opposed to after reperfusion.

## 3. Discussion

Although treatments for heart failure have advanced, the incidence of HF is still rising and new therapies remain an important goal. There is now mounting evidence of a significant role for the UPR in cardiomyocytes during ischemic heart disease. Much remains to be understood with respect to how individual branches of the UPR differentially or synergistically contribute to progression of heart failure and how these pathways differ from requirements of the UPR during development. In addition, the therapeutic and prophylactic potential of modulating the heart is far from complete. Some have suggested that ischemic preconditioning of the heart and activation of the UPR may promote cardiac cell survival. Interesting proofs of principle have been published. For example, *in vitro*, overexpression of ER-stress-induced Grp94 has been shown to inhibit cardiomyocyte necrosis after calcium overload and simulated ischemia [45]. Overexpression of GRP78 has also been shown to have an effect. Forced GRP78 expression inhibited apoptosis in rat ventricular myocytes [57]. Also, preconditioning of H9c2 neonatal cardiomyocytes cells with the ER-stressor tunicamycin has been shown to protect against ATP deletion [58]. These are laudable starts, but much work remains to be done. Future questions remain. For example: What is the effect of chemical chaperones on cardiac stress pathways and cardiac function [59]? Future studies will also be required to dissect the effects of cell-specific deletion of UPR genes in the heart, including cardiomyocytes, myofibroblasts, and inflammatory cells that infiltrate into the myocardium after injury. UPR-targeted therapies may be realized by promoting the cytoprotective function of the UPR in the myocyte. Such an approach may induce UPR-specific ER chaperones and downstream prosurvival pathways that work to enhance cardiac function and prevent cardiomyocyte death.

## Abbreviations

ATF6: Activating transcription factor-6
BIP: Binding immunoglobulin protein
eIF2*α*: Eukaryotic initiation factor 2
ER: Endoplasmic reticulum
ERAD: ER-associated degradation pathway
Grp94: Glucose-regulated protein
HF: Heart failure
IRE-1: Includes inositol-requiring enzyme-1 (IRE-1)
MI: Myocardial infarction
PERK: PKR-like eukaryotic initiation factor 2 kinase
PDI: Protein disulfide isomerase
SR: Sarcoplasmic reticulum
UPR: Unfolded protein response.
